# Retrospective Study of Infections with *Corynebacterium diphtheriae* Species Complex, French Guiana, 2016–2021 

**DOI:** 10.3201/eid3008.231671

**Published:** 2024-08

**Authors:** Mélanie Gaillet, Mélanie Hennart, Vincent Sainte Rose, Edgar Badell, Céline Michaud, Romain Blaizot, Magalie Demar, Luisiane Carvalho, Jean François Carod, Audrey Andrieu, Félix Djossou, Julie Toubiana, Loic Epelboin, Sylvain Brisse

**Affiliations:** Grenoble University Hospital, Grenoble, France (M. Gaillet);; Laboratory TIMC-IMAG, Grenoble Alpes University, Grenoble (M. Gaillet);; Cayenne Hospital Center, Cayenne, French Guiana (M. Gaillet, V. Sainte Rose, C. Michaud, R. Blaizot, M. Demar, F. Djossou, L. Epelboin);; Institut Pasteur, Université Paris Cité, Paris, France (M. Hennart, E. Badell, J. Toubiana, S. Brisse);; National Reference Center for Corynebacteria of the diphtheriae complex, Institut Pasteur, Paris (E. Badell, J. Toubiana, S. Brisse);; French Guiana University, Cayenne (R. Blaizot, M. Demar, F. Djossou, L. Epelboin);; Public Health France, Cayenne (L. Carvalho);; Western Guianese Hospital Center, Saint-Laurent du Maroni, French Guiana (J.F. Carod);; Regional Health Agency, Cayenne (A. Andrieu);; Necker-enfants malades hospital, Paris (J. Toubiana);; INSERM 1424, Cayenne (L. Epelboin)

**Keywords:** bacteria, diphtheria, *Corynebacterium diphtheriae*, Corynebacterium diphtheriae species complex, cutaneous diphtheria, antimicrobial resistance, French Guiana, Amazonia, zoonoses

## Abstract

Human infections with *Corynebacterium* *diphtheriae* species complex (CdSC) bacteria were rare in French Guiana until 2016, when the number of cases diagnosed increased. We conducted an epidemiologic, multicenter, retrospective study of all human CdSC infections diagnosed in French Guiana during January 1, 2016–December 31, 2021. A total of 64 infectious episodes were observed in 60 patients; 61 infections were caused by *C. diphtheriae* and 3 by *C*. *ulcerans*. Estimated incidence increased from 0.7 cases/100,000 population in 2016 to 7.7 cases/100,000 population in 2021. The mean patient age was 30.4 (+23.7) years, and male-to-female ratio was 1.7:1 (38/22). Of the 61 *C. diphtheriae* isolates, 5 tested positive for the diphtheria toxin gene, and all results were negative by Elek test; 95% (61/64) of cases were cutaneous, including the *C. ulcerans* cases. The increase in reported human infections underscores the need to raise awareness among frontline healthcare practitioners to improve prevention.

*Corynebacterium* *diphtheriae* species complex (CdSC; also called cornyebacteria of the *diphtheriae* species complex) include *Corynebacterium diphtheriae* and *C. ulcerans*, 2 potentially toxigenic and highly pathogenic species for humans (*1*). Human corynebacterial infections can be potentially fatal if left untreated (*2*). Risk for infection is higher among immunocompromised persons and socially disadvantaged persons. Socioeconomic challenges in particular are a major concern in French Guiana, a French overseas territory located in northeastern South America and covered by the Amazon Rain Forest (*3*).

The classical clinical manifestation of diphtheria is a pseudomembrane in the upper respiratory airways (tonsils, pharynx, or larynx) that can cause possible fatal airway obstruction. Cutaneous forms are less severe but have been reported more frequently than respiratory forms in recent studies (*4*); those forms play a key role in the transmission of *C. diphtheriae.* In addition, potentially serious systemic nontoxigenic infections have also been observed (*5*). The zoonotic species *C. ulcerans* can also occur as respiratory or cutaneous forms. The severity of the disease is mainly because of the production by toxigenic strains of the diphtheria toxin, which can cause systemic damages, particularly with cardiac and neurologic involvement (*2*).

In French Guiana, diphtheria infection might be suspected by physicians in the presence of a suggestive clinical picture. Diphtheria infection must be confirmed by a standard bacteriological examination. However, clinical diagnosis is not always easy, particularly in the case of the cutaneous form, because of its nonspecific manifestations (*6*) and the many differential diagnoses that exist, such as leishmaniasis or scabies (*7*). The diagnosis can therefore often be made by chance. In accordance with recommendations in France, all bacteriological diagnoses of CdSC must be supplemented by PCR testing for the diphtheria toxin gene, which is performed at the National Reference Centre (NRC) in Paris and, since 2019, at the Cayenne Hospital Center (CHC) for all of French Guiana. Depending on the clinical manifestations and the species and presence of the toxin, clinical management of diphtheria involves respiratory or wound isolation, antibiotic therapy, and vaccine update, as well as screening and management of contact cases (*8*). If the toxin is present, administering diphtheria antitoxin should be considered rapidly, depending on the symptoms (a supply is available at CHC) (*8*).

However, clinical management is complex in French Guiana because of the challenges in access to care and prevention for populations living in remote areas of the territory (*9*). Furthermore, availability of healthcare services remains inadequate, particularly in those remote areas (*10*). Although 80% of the population lives in the coastal zone, where healthcare services are concentrated (*10*,*11*), ≈60,000 persons live in isolation along the rivers and in areas of the Amazon Rain Forest in the interior of the territory (*3*).

Immunization is the most effective approach to prevent severe infections (*5*). According to available estimates, the diphtheria vaccination coverage rate for persons <18 years of age in French Guiana was 63.4% in 2014. Given the absence of a large-scale vaccination campaign in the territory during 2014–2021 and the delay in vaccination during the COVID-19 pandemic, that rate will likely remain low (*12*,*13*).

Cases of corynebacterial human infections were rare in French Guiana until 2016; only 2 cases, both from nontoxigenic *C. diphtheriae* strains, an unspecified bacteremia form and an endocarditis, were described in this territory (*14*,*15*). Since 2016, however, physicians and laboratories have reported an abnormally high number of these infections compared with previous years. To learn more about this emergence, our main objective was to describe sociodemographic, clinical, and microbiological characteristics of those cases. The secondary objectives were to estimate the annual incidence of cases, to study the resistance phenotypes of the bacterial isolates, and to study the genetic diversity of those isolates and potential links between cases.

## Methods

### Study Design and Settings

**Figure 1:**
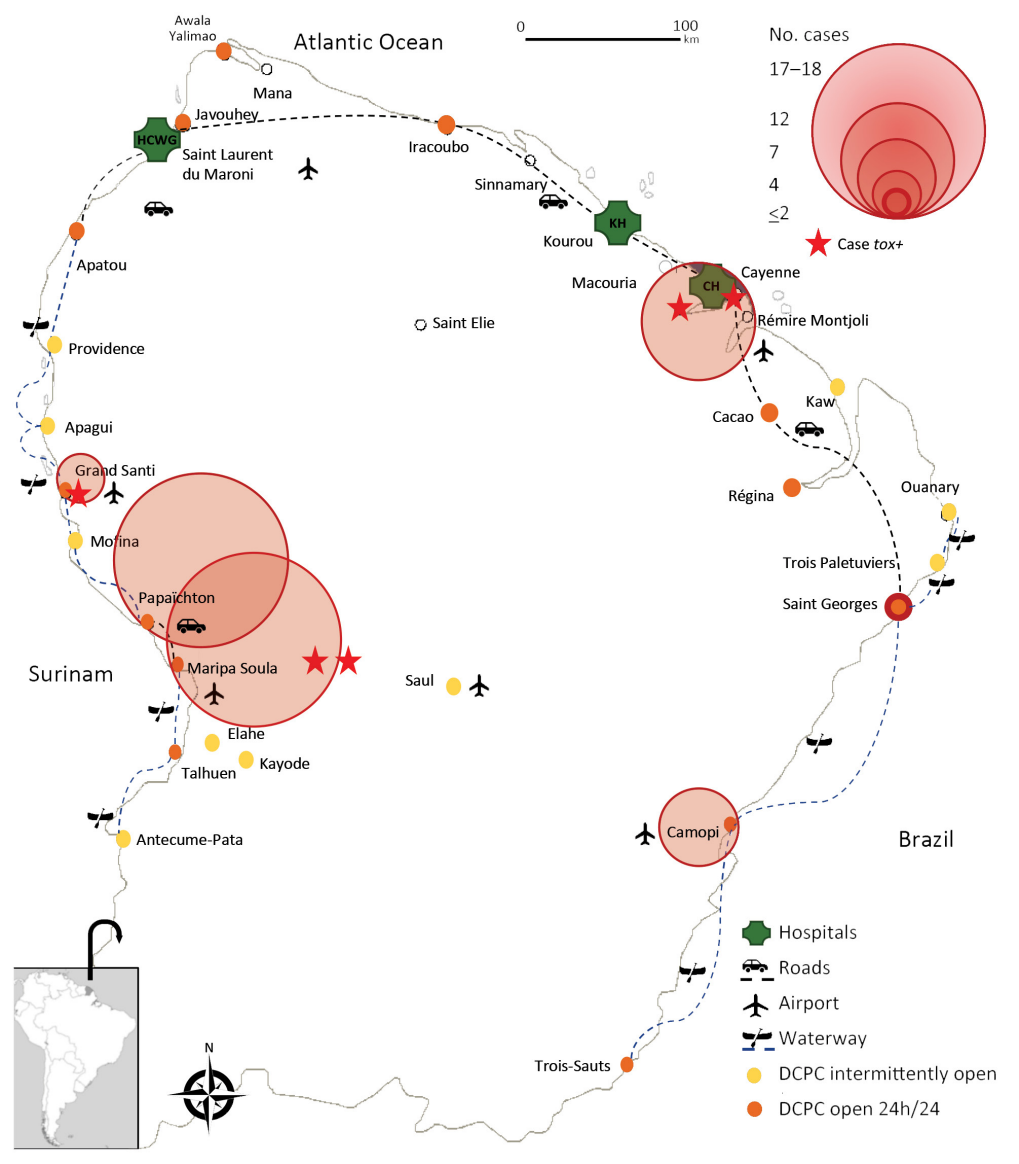
Geographic distribution of 64 cases of *Corynebacterium* *diphtheriae* species complex infections, French Guiana, 2016–2021. Inset map shows location of French Guiana in South America. DCPC, Delocalized Centers for Prevention and Care.

We conducted a retrospective and multicenter study in French Guiana, which covers an area of 83,846 km^2^ and neighbors Brazil and Suriname. The private healthcare sector in French Guiana is limited; healthcare services are provided mainly by 3 hospital centers. CHC and Western Guianese Hospital Center (WGHC) are the 2 major healthcare centers (Figure 1). A total of 16 Delocalized Centers for Prevention and Care (DCPCs), which are a hospital department of CHC (i.e., sharing the same medical tools and software), oversee the primary care for persons in the Amazon areas of the territory. All samples collected in the DCPCs were sent to CHC, except samples from the DCPCs of Grand Santi, Apatou, Javouhey, and Awala Yalimapo, which were sent to WGHC by agreement.

### Sociodemographic and Clinical Data Collection

We extracted all cases with a bacterial isolate belonging to the CdSC, laboratory diagnosed in CHC or WGHC during January 1, 2016–December 31, 2021, using HEXALIS results-reporting software (AGFA Healthcare). We arbitrarily considered positive samples for the same patient as 2 distinct episodes when they were collected >6 months apart and after clinical cure of the first episode or negative bacteriological control after >1 month of well-managed treatment.

We extracted patient sociodemographic and clinical data using the medical monitoring software (Cora Systems) for CHC and WGHC (including DCPC). We excluded patients without any clinical information found. Patients who objected to the use of their medical data in this study were secondarily excluded. We treated patient data that were not available in the medical records as unavailable data. For data describing patient progress, we considered patients who did not attend scheduled consultations as lost to follow-up (and not as unavailable data).

### Bacterial Isolation, Identification, and Toxigenicity and Antimicrobial Susceptibility Testing

We identified and cultivated bacteria at CHC and WGHC on Columbia horse blood agar plate (bioMérieux) for 24 hours at 35°C–37°C. We performed bacterial identification by using Bruker matrix-assisted laser desorption/ionization time-of-flight mass spectrometry. We routinely sent all CdSC isolates to the NRC at the Institut Pasteur of Paris for further analysis.

At NRC, isolates were grown and purified on Tinsdale agar and characterized for pyrazinamidase, urease, nitrate reductase, use of maltose, and glycogen fermentation (*16*). We used a 4-plex real-time PCR to detect the diphtheria toxin gene and confirm species (*17*). We assessed the phenotypic production of the toxin using a modified Elek test (*18*). We performed antimicrobial susceptibility testing by disc diffusion (Bio-Rad Laboratories) and used E-test for MIC determination if deemed resistant on the basis of disk diffusion (Appendix). 

### Genome Sequencing and Phylogenetic Analyses

We performed genomic analyses at NRC using Illumina technology and de novo assembly as previously described (*16*,*19*). We conducted the search for resistance genes and *tox* gene integrity using diphtOscan (*20*). We defined multilocus sequence type (MLST) and core-genome MLST (cgMLST) genotypes using the Institut Pasteur CdSC database (https://bigsdb.pasteur.fr/diphtheria). For *C. diphtheriae* isolates, we defined the sublineages (500 mismatches) and genetic clusters (25 mismatch threshold) groupings as previously proposed (*19*) (Appendix).

### Statistical Analysis

We performed statistical analyses using Microsoft Excel and RStudio version 02.3 software. We expressed qualitative variables as numbers and percentages and distribution of quantitative variables as means and SDs. We calculated estimates of annual incidence per 100,000 inhabitants on the basis of demographic data published annually by the French National Institute of Statistics and Economic Studies during 2016–2021 (*21**−**26*) (Appendix).

### Ethics Approval

In conformity with French legislation, this retrospective study not involving human persons adheres to the Reference Methodology MR-004, with CHC’s compliance commitment dated December 21, 2021. A privacy impact analysis was conducted; a study summary is available on the Health-Data-Hub (no. F20220825152116). Its legal basis is a public interest mission. Data were sourced from routine care patient medical files and anonymized. Regulatory steps were taken to inform patients and allow refusal (Appendix). This approach was conducted in compliance with regulations in Europe (https://www.cnil.fr/fr/reglement-europeen-protection-donnees).

## Results

### Included Bacterial Isolates and Associated Clinical Cases

**Figure 2:**
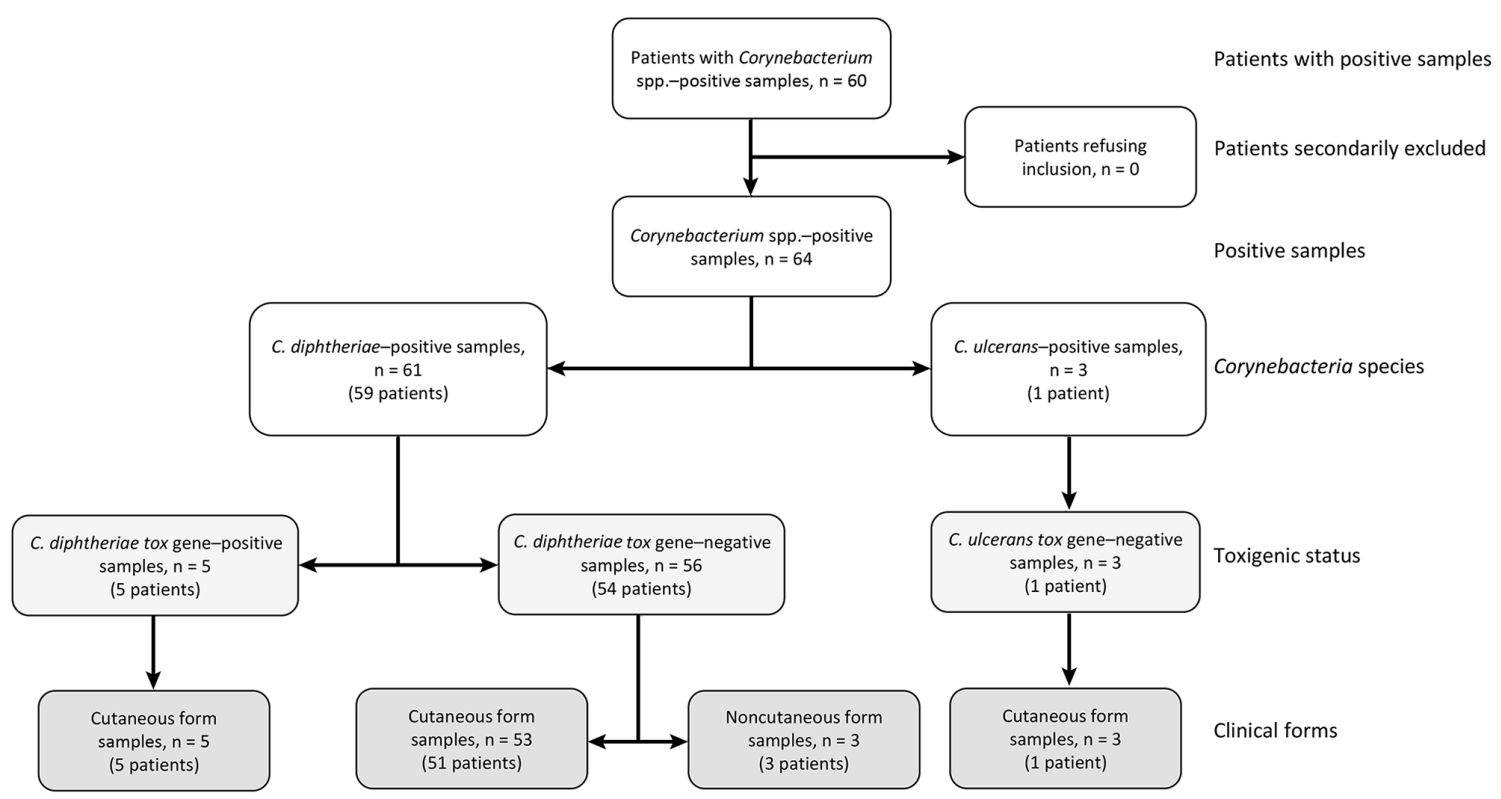
Flowchart of cases included in retrospective study of infections with *Corynebacterium* *diphtheriae* species complex, French Guiana, 2016–2021.

For the study period, we identified 64 cases of bacterial isolates of CdSC isolated from 60 patients. No patients objected to the use of their data for the study, and none were excluded (Figure 2). We found 61 cases of *C. diphtheriae*, corresponding to 58 cutaneous forms and 3 noncutaneous forms (1 superinfection of chronic nasal mucosal disease, 1 respiratory infection, and 1 endocarditis), and 3 cases of *C. ulcerans*, all of which were cutaneous forms. Of the 60 patients, 2 had 2 positive *C. diphtheriae* samples each, corresponding to unrelated cases of cutaneous infection, and 1 patient had 3 positive *C. ulcerans* samples, corresponding to 3 different cases.

A total of 5 *C. diphtheriae* cases were *tox-*positive by quantitative PCR (qPCR) (8%; 5/64). However, they did not produce the diphtheria toxin; they were all negative by Elek’s test. Those 5 cases thus correspond to nontoxigenic *tox* gene–bearing isolates.

### Evolution of Estimated Incidence during Study Period

**Figure 3:**
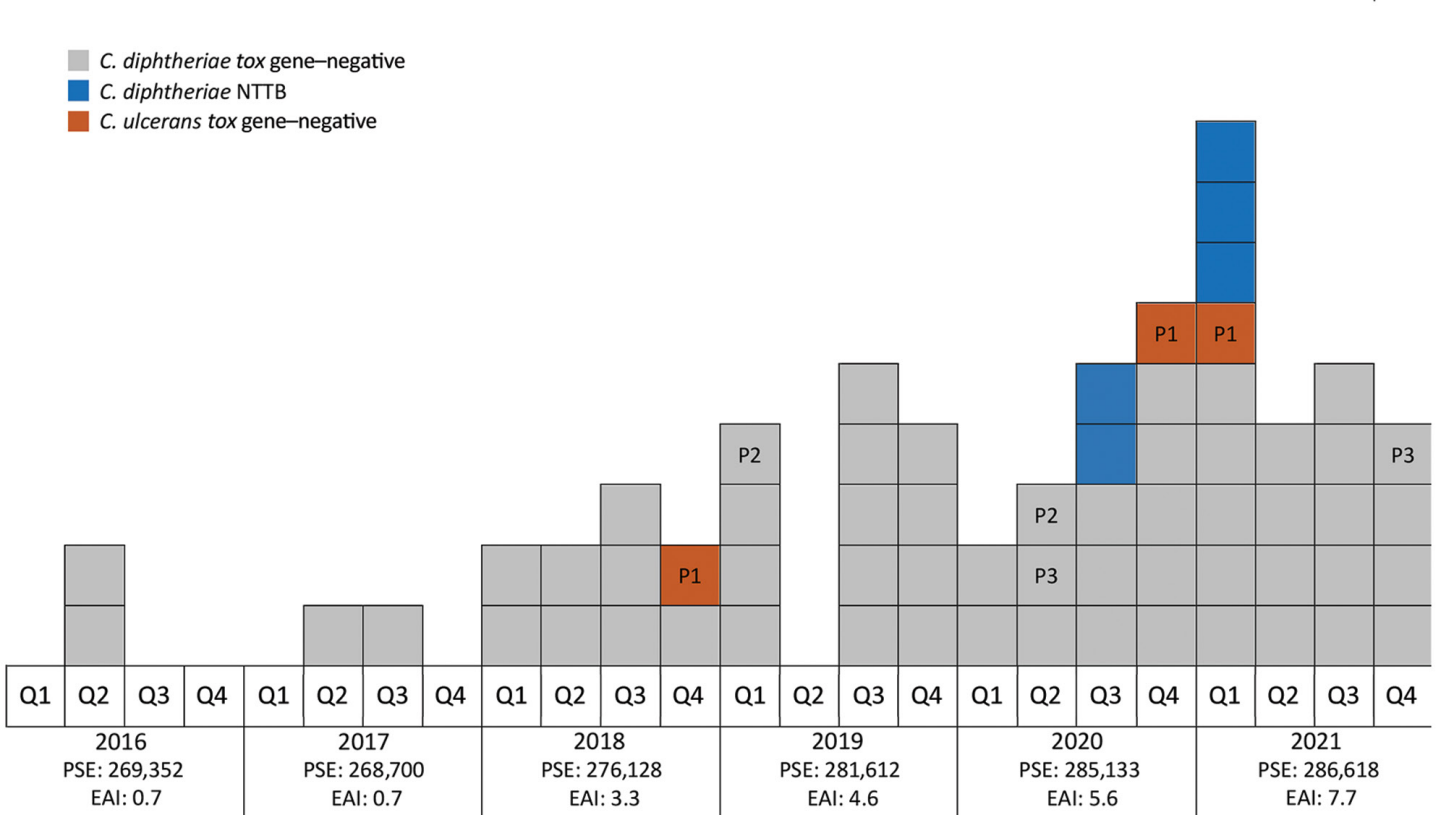
Temporal distribution of isolates corresponding to 64 clinical episodes of infection with *Corynebacterium* *diphtheriae* species complex in French Guiana, 2016–2021. Each box indicates 1 case; patient number labels (e.g., P1) indicate multiple cases in the same patient. EAI, estimated annual incidence (cases/100,000 population); NTTB, nontoxigenic *tox*-gene–bearing; PSE, population size estimation; Q, quarter.

The number of annual cases has risen steadily. Only 2 cases were diagnosed in 2016 and 2017, but in 2022, the number of annual cases reached 22. The estimated incidence has risen from 0.7 cases/100,000 inhabitants/year in 2016 to 7.7 cases/100,000 inhabitants/year in 2021 (Figure 3).

### Epidemiology, Sociodemographics, and Medical History of Patients

In total, 80% (51/64) of the cases in our study were diagnosed in a DCPC by initial bacteriological sampling, whereas the others were mainly geographically dispersed (Figure 1). Mean patient age was 30.4 (+23.7) years (interquartile range 10.5–49.5) the male-to-female ratio was 1.7:1 (38/22). Of the 55 patients for whom information was available, 19 (35%) did not have social security coverage at the time of care, and 3 (5%) were homeless.

Of 58 patients with available data, 6 (10%) had a history of relative immunosuppression (diabetes, n = 3; alcoholic cirrhosis, n = 2; panhypopituitarism, n = 1). In addition, 10 (17%) were alcoholics, smokers, or polydrug addicts; 3 (5%) had a psychiatric pathology; and 8 (14%) had a chronic skin pathology that predisposed them to wounds or superinfections. Of 44 patients with available vaccination data, 29 (66%) were up-to-date with diphtheria vaccination at the time of infection (in accordance with French Public Health requirements for diphtheria) (*27*).

### Clinical Description of Cases Caused by *C. diphtheriae*

Among the 61 cases caused by *C. diphtheriae*, 58 were cutaneous infections and 3 noncutaneous infections. Of the 58 cutaneous infections, the infection occurred on an existing wound in 23 (40%) persons: 6 occurred in a chronic skin lesion (lasting >6 weeks) and 17 occurred in an acute lesion (lasting <6 weeks). The infections mainly involved the lower limbs (62%, 36/58), followed by the head (12%, 7/58), the abdomen (7%, 4/58) and the upper limbs (2%, 1/58); 17% of cases extended to >2 areas of the body (10/58). In 57% (33/58) of cases, several lesions were present; in 25 cases, fibrinous involvement was described (56%, 25/45; data were not available for 13 cases). One patient, in whom 2 cases of cutaneous infection were present, had a background of immunosuppression; those 2 cases occurred on a chronic cutaneous lesion.

Data regarding clinical management were available for 52 cases. For 6 (12%) cases, treatment consisted only of simple dressing care. The 46 (88%) other cases were also treated by antibiotics; 40 were treated with amoxicillin (16 for 14 days, 16 for <10 days; data were not available for 8 cases), 2 were treated with azithromycin for 3 days, and 4 were treated with ciprofloxacin, pristinamycin, or ceftriaxone. One patient with multiple abscesses underwent surgery. The 5 patients with *tox-*positive isolates had cutaneous infection without toxic manifestations. No patients received diphtheria antitoxin while results of the qPCR were pending because they had no signs of severity attributable to toxin expression. For the 58 patients with *C. diphtheria* cutaneous infections, the outcome was favorable for 22 persons, 4 were lost to follow-up, and information was missing for 25. The clinical outcome was described as unfavorable for 7 patients (i.e., 1 patient died, 2 had a recurrence after 1 month, ulcerations were described as persistent despite well-managed treatment for 2 others, and details were not available for 2 more). For 18 cases (44%, 18/41 with available data among the 58 *C. diphtheriae* cutaneous cases), an investigation was conducted into the case.

For the 3 noncutaneous infections, the first patient (68 years of age) had a lower respiratory infection; *Staphylococcus aureus*, *Pseudomonas aeruginosa*, and *Corynebacterium diphtheriae* were also found in the bronchial aspirate. The patient died of septic shock caused by *Klebsiella pneumoniae*. The second patient, a 2-year-old child with a polymalformative syndrome, had a diagnosis of endocarditis to *C. diphtheriae* (confirmed by blood sample and secondarily confirmed by cardiac biopsy). The third patient (72 years of age) experienced a nasal mucosal form (superinfection of a chronic injury confirmed by mucus sampling). For those last 2 patients, details on the nature of the treatment were not available, but the clinical outcome was favorable. The diphtheria vaccination status of those patients was unknown.

### 3 Cutaneous Cases Caused by *C. ulcerans* in 1 Patient

One patient (74 years of age), who did not live with pets, was up-to-date on diphtheria vaccination, and had an underlying chronic skin wound (lymphatic filariasis sequelae), experienced 3 unrelated cases at 1-year intervals; eradication was monitored by samples collected 2 months after acute and treated cases. He had several lesions with fibrinous involvement located in the lower limbs (2 cases) and in upper limbs (1 case). Treatment consisted of amoxicillin for the first infection (unknown duration) and trimethoprim/sulfamethoxazole (for 10 days) for the second one; treatment for the last infection was not specified. The clinical course was described as good for all infections. Investigation into the case was not documented. The 3 *C. ulcerans* isolates belonged to the same genotype (sequence type [ST] 719) and were almost identical at the genomic sequence level (Appendix Figure).

### Description of Coinfections

Of the 64 samples included, 61 (95%) were associated with bacterial co-infections. Among those 61 co-infections, 44 (69%) isolates were associated with a *Streptococcus* sp., 45 (70%) with *S*. *aureus*, and 31 (48%) with both of those 2 species; 15 (23%) were co-infected with another bacterium. The 3 non–co-infected cases corresponded to a blood culture sample and 2 cutaneous forms.

### Genomic Epidemiology of Isolates

**Figure 4:**
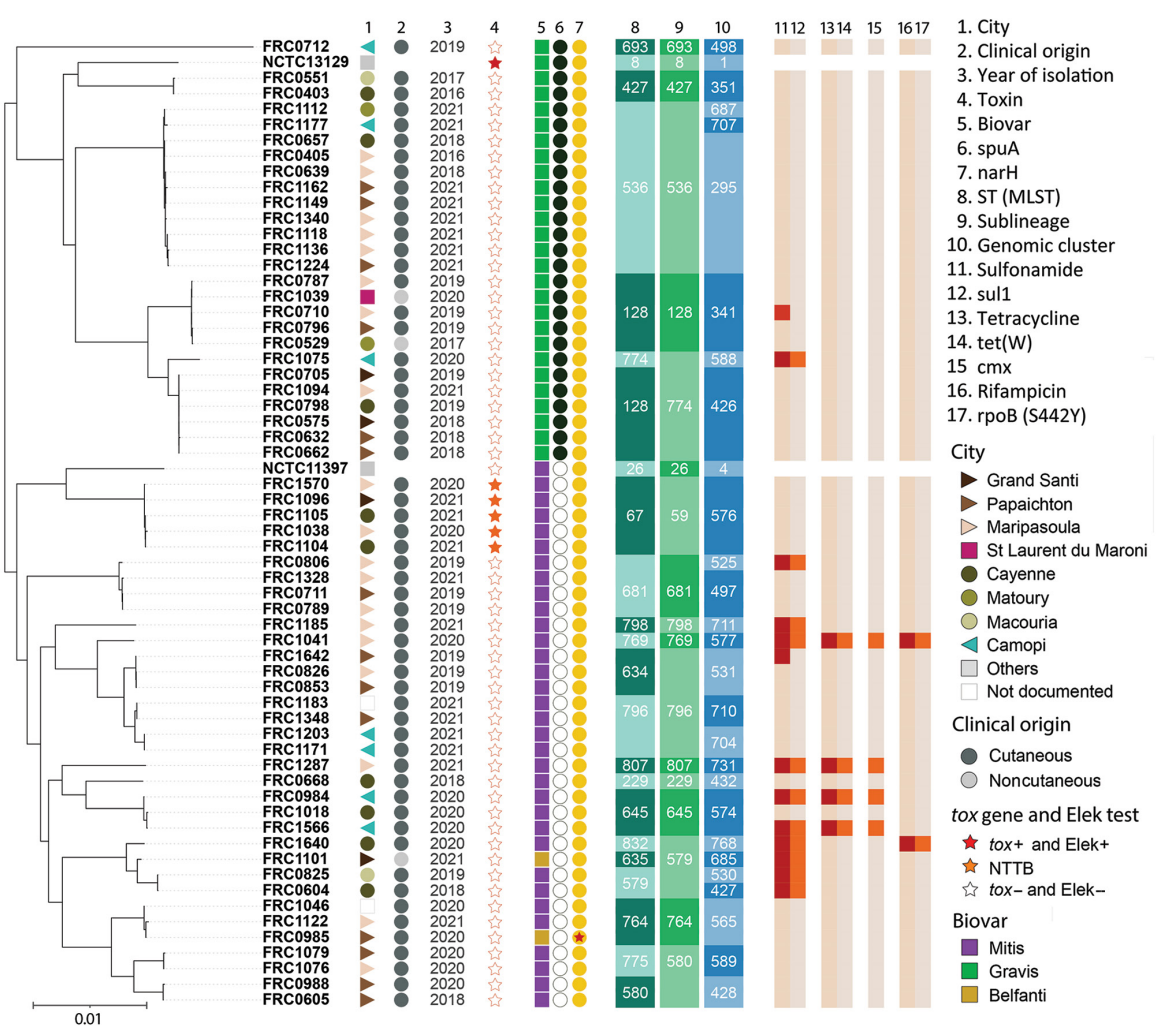
Diversity of *Corynebacterium diphtheriae* isolates in retrospective study of infections with *C. diphtheriae* species complex, French Guiana, 2016–2021. Star in column 7 indicates narH gene was not complete. MLST, multilocus sequence type; ST, sequence type.

We studied the genomic diversity of 63 sequenced isolates (1 *C. diphtheriae* isolate could not be sequenced). The phylogenetic structure of the *C. diphtheriae* isolates from French Guiana formed 2 main lineages (Figure 4); 26 isolates corresponded to biovar Gravis (and its *spuA* marker gene) and 32 corresponded to biovar Mitis (*16*). Two isolates were biovar Belfanti (nitrate negative); for 1 of those, a disruptive mutation on the *narH* gene involved in nitrate reduction was found.

For *C. diphtheriae* isolates, sublineage-level classification showed 16 distinct sublineages (SL; defined using the 500 cgMLST mismatch threshold) (Figure 4). The most frequent SL was SL536 (6.6%, 11/63; all ST536). The other SLs had on average 3.3 isolates (SD 2.1). On the finer classification level, 26 genomic clusters were identified (defined using the 25 cgMLST mismatch threshold). One genomic cluster, GC295, was collected 9 times and belongs to the predominant SL536 (Figure 4). The 5 *tox*-positive isolates (but nontoxigenic because of a stop codon) belonged to the same sublineage and genomic cluster (ST67, all biovar Mitis).

When reanalyzing 1,350 global *C. diphtheriae* isolates (*20*), we found only 3 sublineages of this study were also observed outside of French Guiana: in metropolitan France, Brazil, or Malaysia. At the genomic cluster level, only 1 French Guiana genotype (GC341) was also observed in metropolitan France, consistent with this patient having recently traveled there. Most isolates (41/63) were susceptible to all antimicrobial drugs tested (Appendix).

## Discussion

We investigated 64 human cases of corynebacterial infections in 60 patients observed during 2016–2021 in French Guiana and found none were toxigenic. Most cases were cutaneous, although 1 case of endocarditis was observed. Cases were diagnosed mainly in the DCPCs located throughout the remote Amazon territories, where care can be difficult to perform. The increase in cases during this period raises public health concerns, and several factors should be considered.

Bacteriological techniques for identifying isolates evolved in French Guiana in 2016 with the introduction of matrix-assisted laser desorption/ionization time-of-flight mass spectrometry. This tool has probably contributed to improved identification of corynebacterial infections. In addition, the emergence of cases constituted a health signal. As a result, beginning in late 2018, healthcare professionals in French Guiana were taught by the territory’s infectiologists how to diagnose and manage diphtheria, in line with current national recommendations (*8*). That training might have contributed to improved detection of cases by clinicians. However, the increase in cases during 2018 when that additional measure had not yet been deployed argues in favor of an actual increase in incidence.

Several factors suggest that cases were still underdiagnosed and therefore underestimated. First, the aspecific nature of cutaneous forms of infection, as confirmed in this study, means that clinicians might have missed diagnoses (*6*). In addition, national recommendations in France for the management of cutaneous wounds do not recommend bacteriological sampling (*28*), leading to a probable lack of screening for cutaneous forms. Finally, in 2020 and 2021, the COVID-19 pandemic affected access to healthcare in French Guiana because of repeated lockdowns and reorientation of medical care toward health crisis management (*9*). The number of cases was therefore probably underestimated during this period, despite the observed increase in incidence.

As discussed previously, cutaneous forms of CdSC infections can very easily go unnoticed, which raises several questions. The probable antimicrobial therapy recommended for managing skin wounds is a short treatment, usually <7 days, targeting *Staphylococcus* and *Streptococcus* (*28*). The antimicrobial agents used, mainly amoxicillin/clavulanic acid, also correspond to the first-line treatment for diphtheria. However, the duration (7 days vs. 14 days as recommended for diphtheria infections) (*8*) is insufficient to obtain optimal clearance (*29*,*30*). Furthermore, use of an antibiogram is crucial because of emerging antimicrobial resistance (*12*,*16*). We identified 4 multidrug-resistant isolates (including the same genomic context). Therefore, lack of diagnosis and appropriate medical care exposes the risk for inadequate treatment, which can lead to unfavorable wound progression, risk for recurrence, and community spread of the pathogen (*23*). Last, the lack of contact patient screening also likely affected risk for transmission (*12*,*24*,*25*). Here, we observed 20 genomic clusters (with >1 isolate), distributed in time or space, demonstrating active transmission in the population. Furthermore, some inhabitants of French Guiana, particularly those living in remote areas, follow a cross-border lifestyle and are mobile in the territory, which, in addition to making care more complex, can contribute to spread (*26*).

All of those factors call for increased vigilance and a review of recommendations for screening and managing skin wounds, which should be adapted to the specific epidemiologic characteristics of French Guiana. In addition, taking better account of the risk for diphtheria when evaluating upper respiratory and systemic infections in French Guiana is key. Building and maintaining awareness of corynebacterial infections among healthcare providers throughout the territory is essential. In 2019, French Guiana began deploying mobile public health teams within the territory, but that effort was focused on the fight against the pandemic during 2020–2021 and could not fully contribute to the management of diphtheria cases. Those mobile health teams will provide valuable assistance in the management of patients who are farthest from care and will conduct investigations around cases using the go-to approach (i.e., teams going to the patient to provide care). Because of lacking human resources, qPCR cannot always be conducted at the CHC, extending the time taken to obtain results by 3 to 5 days after strains have been sent to Paris. Increasing the capacity of CHC to carry out routine toxin qPCR is therefore necessary. Last, increasing diphtheria vaccination coverage in French Guiana is key to combatting this problem effectively. Insufficient diphtheria vaccination coverage exposes the population at increased risk for circulation of toxigenic strains which could find a favorable niche. There are migratory flows in French Guiana from countries where diphtheria epidemics have been described (Brazil, Haiti, Dominican Republic) and where vaccination coverage is low (*31*). However, *C. diphtheriae* appears to have a phylogeographic structure represented by area-specific variants, and almost all isolates described in French Guiana have not been described elsewhere.

This study’s first limitation is that, because of its retrospective nature, a substantial amount of information is lost. Moreover, data collection only concerned the 2 main hospitals in French Guiana and did not include private laboratories or Center Hospital of Kourou, limiting its exhaustiveness and impact estimates, even though no other cases were transmitted to the NRC during the study period.

In conclusion, nontoxigenic corynebacterial cutaneous infections have been increasingly diagnosed in French Guiana since 2016. A few severe forms have been described (i.e., endocarditis, surgical lesion). Given low vaccination coverage and the presumed chains of transmission, toxigenic isolates could find a favorable environment if introduced from other countries. These considerations call for regular training courses to raise awareness among frontline workers. In addition, reinforcing and adapting diagnostic and management recommendations in French Guiana is essential. Infections with members of the CdSC remains a public health issue, and increasing awareness among clinicians worldwide is necessary.

Appendix Additional information about retrospective study of infections by Corynebacteria of *diphtheriae* species complex, French Guiana, 2016–2021.
