# Supplementary material for: Retrospective Study of Infections with Corynebacterium diphtheriae Species Complex, French Guiana, 2016–2021
Source: Emerg Infect Dis. 2024 Aug;30(8):1542–51. doi: 10.3201/eid3008.231671 (PMC11286053; doi:10.3201/eid3008.231671)
Supplement: Appendix — Additional information about retrospective study of infections by Corynebacteria of diphtheriae species complex, French Guiana, 2016–2021. [file 23-1671-Techapp-s1.pdf]

Article DOI: https://doi.org/10.3201/eid3008.231671
EID cannot ensure accessibility for supplementary materials supplied by authors. Readers who have difficulty accessing supplementary content should contact the authors for assistance.

Retrospective Study of Infections with *Corynebacterium diphtheriae* Species Complex, French Guiana, 2016–2021

Appendix

Genome sequencing

Further genomic analyses were performed by the NRC for Corynebacteria of the *Corynebacterium* diphtheriae complex in Institut Pasteur, Paris. Isolates were retrieved from −80°C storage and plated on tryptose-casein soy agar for 24 to 48 h. A small amount of bacterial colony biomass was resuspended in a lysis solution (20 mM Tris-HCl [pH 8], 2 mM EDTA, 1.2% Triton X-100, and lysozyme [20 mg/ml]) and incubated at 37°C for 1 h DNA was extracted with the DNeasy Blood&Tissue kit (Qiagen, Courtaboeuf, France) according to the manufacturer’s instructions. Genomic sequencing was performed using a NextSeq500 instrument (Illumina, San Diego, CA) with a 2 × 150-nt (nt) paired-end protocol following Nextera XT library preparation (*1*).

For de novo assembly, paired-end reads were clipped and trimmed using AlienTrimmer v0.4.0 (*2*), corrected using Musket v1.1 (*3*), and merged (if needed) using FLASH v1.2.11 (*4*). For each sample, the remaining processed reads were assembled and scaffolded using SPAdes v3.12.0.

Phylogenetic analyses

For the phylogenetic analyses, Panaroo v1.2.3 was used to generate from the assembled genomic sequences, a core genome used to construct a multiple sequence alignment (cg-MSA). The genome sequences were first annotated using prokka v1.14.5 with default parameters, resulting in GFF files. Protein-coding gene clusters were defined with a threshold of 70% amino acid identity, and core genes were concatenated into a cg-MSA when present in 95% of genomes. IQtree version 2 was used to build a phylogenetic tree based on the cg-MSA, with the

best fitting model GTR+F+I+G4. The tree was constructed from 1,903 core genome loci, for a total alignment length of 1,927,340 bp (77% of NCTC13129 genome length, of 2,488,635 bp).

cgMLST and nomenclature of sublineages

The MLST and cgMLST genotypes (cgST) were defined using the Institut Pasteur *C. diphtheriae* species complex database at https://bigsdb.pasteur.fr/diphtheria. Only for the *C. diphtheriae* isolates, the nomenclature 'sublineages' (SL; 500 mismatches) and 'genetic clusters' (GC; 25 mismatch threshold) were defined as previously proposed by Guglielmini *et al.* (*5*). *C. diphtheriae* isolates can be classified into 7-gene MLST sequence types and into phylogenetic sublineages, which represent deep phylogenetic subdivisions of the population structure of this species that are highly concordant with ST classifications. GC are much narrower genetic subdivisions of SLs that have been defined as groups of isolates that have, among themselves, genetic distances compatible with outbreaks or recent transmission. For *C. ulcerans*, we used the cgMLST scheme developed recently and incorporated into the BIGSdb-Pasteur diphtheria platform (https://bigsdb.pasteur.fr/diphtheria/).

Calculation of impact estimates

[(number of CsDS cases diagnosed in the year "n" x 100,000)

/ number of inhabitants of French Guiana in the year "n"]

We took the number of cases per year between 2016 and 2021 described in Figure 3 as the numerator as follows:

- 2016: number of cases: 2
- 2017; number of cases: 2
- 2018; number of cases: 9
- 2019; number of cases: 13
- 2020; number of cases: 16
- 2021; number of cases: 22

We took as denominator, the estimates of Guyanese population size by year, between 2016 and 2021, published by French National Institute of Statistics and Economic Studies (or Institut National de Statistiques et Etudes Economiques (*6–11*), as follows:

- 2016: 269352 inhabitants;
- 2017; 268700 inhabitants;
- 2018; 276128 inhabitants;
- 2019; 281612 inhabitants;
- 2020; 258133 inhabitants;
- 2021; 286618 inhabitants;

Ethical Approval (Details)

In conformity with French legislation, this retrospective study, not involving human persons, adheres to the "Reference Methodology" MR-004, with CH's compliance commitment dated 21/12/2021. A privacy impact analysis was conducted, a study summary is available on the Health-Data-Hub (NN° F20220825152116). Its legal basis is a public interest mission. Data were sourced from routine care patient medical files and anonymised. Regulatory steps were taken to inform patients and allow them to refuse. We tried to reach them by telephone, and then sent a postal letter to non-respondents. Collective information was posted in the care areas known to the people, in their mother tongue, with the support of health mediators. For minors, non-opposition from both legal guardians was required. Deceased participants who hadn't objected during their lifetime were included by default. This approach was carried out in compliance with European regulations (https://www.cnil.fr/fr/reglement-europeen-protection-donnees).

Antimicrobial susceptibility phenotypes and associated genotypes

The isolates were mostly susceptible to all antimicrobials tested (https://docs.google.com/spreadsheets/d/1dK5cCe496bdzQExL4wJFLNY-sbh2hEW4/edit?usp = sharing&ouid = 109112878121272539449&rtpof = true&sd = true).

Genomic analysis of antimicrobial resistance genes showed the presence of antimicrobial resistance genes or mutations against four classes of antimicrobial agents (Table S2): sulfonamide (n = 11), tetracycline (n = 7), phenicol (n = 4) and rifamycin (n = 2). Genes *sul1* *(*sulphonamide resistance), and *tet(W)* and *tet(A)* (tetracycline resistance) were found and associated with cognate phenotypic resistance. The *cmx* gene was also found in two isolates, but this agent was not tested phenotypically. For rifamycin, the two phenotypically resistant isolates had a point mutation in the *rpoB* gene leading to the S442Y amino-acid change. Four isolates were multidrug resistant (acquired resistance to ≥3 classes of drugs), carrying at the same time *sul1*, *tet(W)* and *cmx*. They belonged to three different phylogenetic sublineages, implying genetic transfer of the resistance genes.

References

1. Hennart M, Panunzi LG, Rodrigues C, Gaday Q, Baines SL, Barros-Pinkelnig M, et al. Population genomics and antimicrobial resistance in Corynebacterium diphtheriae. Genome Med. 2020;12:107. PubMed https://doi.org/10.1186/s13073-020-00805-7

2. Criscuolo A, Brisse S. AlienTrimmer: a tool to quickly and accurately trim off multiple short contaminant sequences from high-throughput sequencing reads. Genomics. 2013;102:500–6. PubMed https://doi.org/10.1016/j.ygeno.2013.07.011

3. Liu Y, Schröder J, Schmidt B. Musket: a multistage k-mer spectrum-based error corrector for Illumina sequence data. Bioinformatics. 2013;29:308–15. PubMed https://doi.org/10.1093/bioinformatics/bts690

4. Magoč T, Salzberg SL. FLASH: fast length adjustment of short reads to improve genome assemblies. Bioinformatics. 2011;27:2957–63. PubMed https://doi.org/10.1093/bioinformatics/btr507

5. Guglielmini J, Hennart M, Badell E, Toubiana J, Criscuolo A, Brisse S. Genomic epidemiology and strain taxonomy of *Corynebacterium diphtheriae*. J Clin Microbiol. 2021;59:e0158121. **PMID 34524891**

6. Raimbaud B. Population census in French Guiana: 269,352 inhabitants on 1 January 2016 [in French] [cited 2024 Apr 11]. https://www.insee.fr/fr/statistiques/3679865

7. Jeanne-Rose M. 268,700 inhabitants on 1 January 2017: population census in French Guiana [in French] [cited 2024 Apr 11]. https://www.insee.fr/fr/statistiques/4271842

8. Reif X, Chanteur B. Population census in French Guiana: 276,128 inhabitants on 1 January 2018 [in French] [cited 2024 Apr 11]. https://www.insee.fr/fr/statistiques/5005684

9. Douriaud C, Reif X. Population census in French Guiana: 281,678 inhabitants on 1 January 2019 [in French] [cited 2024 Apr 11]. https://www.insee.fr/fr/statistiques/6012651

10. Douriaud C. 285,133 inhabitants in French Guiana on 1 January 2020 [in French] [cited 2024 Apr 11]. https://www.insee.fr/fr/statistiques/6681450

11. Douriaud C. 286,618 inhabitants in French Guiana on 1 January 2021 [in French] [cited 2024 Apr 11]. https://www.insee.fr/fr/statistiques/7739154

Appendix Table. Antimicrobial agents and zone diameters interpretative breakpoints used

Antimicrobial agent	Abbreviation	S	I	R	Interpretation criteria	Comment
Amoxicillin	AMX	> = 23	16–22.99	<16	CA-SFM, 2013	
Azithromycin	AZM	> = 22	17–21.99	<17	CA-SFM, 2013	
Cefotaxime	CTX	> = 50	15–49.99	<15	EUCAST 2023	
Ciprofloxacin	CIP	> = 50	24–49.99	<24	EUCAST 2023	
Clarithromycin	CLR	> = 22	19–21.99	<19	CA-SFM, 2013	
Clindamycin	CLI	> = 15		<15	EUCAST 2023	Only *C. diphtheriae*, not *C. ulcerans*
Erythromycin	ERY	> = 24		<24	EUCAST 2023	
Gentamicin	GEN	> = 23		<23	CA-SFM/EUCAST, 2019	
Imipenem	IPM	> = 24	17–23.99	<17	CA-SFM, 2013	
Kanamycin	KAN	> = 17	15–16.99	<15	CA-SFM, 2013	
Linezolid	LIN	> = 25		<25	EUCAST 2023	
Oxacillin	OXA	> = 20		<20	CA-SFM, 2013	
Penicillin (10 IU)	PEN_1_IU	> = 50	12–49.99	<12	EUCAST 2023	
Penicillin (1 IU)	PEN_10_IU	> = 29	18–28.99	<18	CA-SFM, 2013	
Pristinamycin	PT	> = 22	19–21.99	<19	CA-SFM, 2013	
Rifampin	RIF	> = 24		<24	CA-SFM, 2013	
Spiramycin	SP	> = 24	19–23.99	<19	CA-SFM, 2013	
Sulfonamide	SUL	> = 17	12–16.99	<12	CA-SFM, 2013	
Tetracycline	TET	> = 24		<24	EUCAST 2023	Interpretation is applicable to doxycycline
Trimethoprim	TMP	> = 16	12–15.99	<12	CA-SFM, 2013	
Trimethoprim-Sulfamethoxazole	SXT	> = 23		<23	EUCAST 2023	
Vancomycin	VAN	> = 17		<17	CA-SFM, 2013	
Meropenem	MER	> = 24		<24	EUCAST 2023	
Fosfomycin	FOS	> = 14		<14	CA-SFM, 2013	
Moxifloxacin	MXF	> = 24	21–23.99	<21	CA-SFM, 2013	

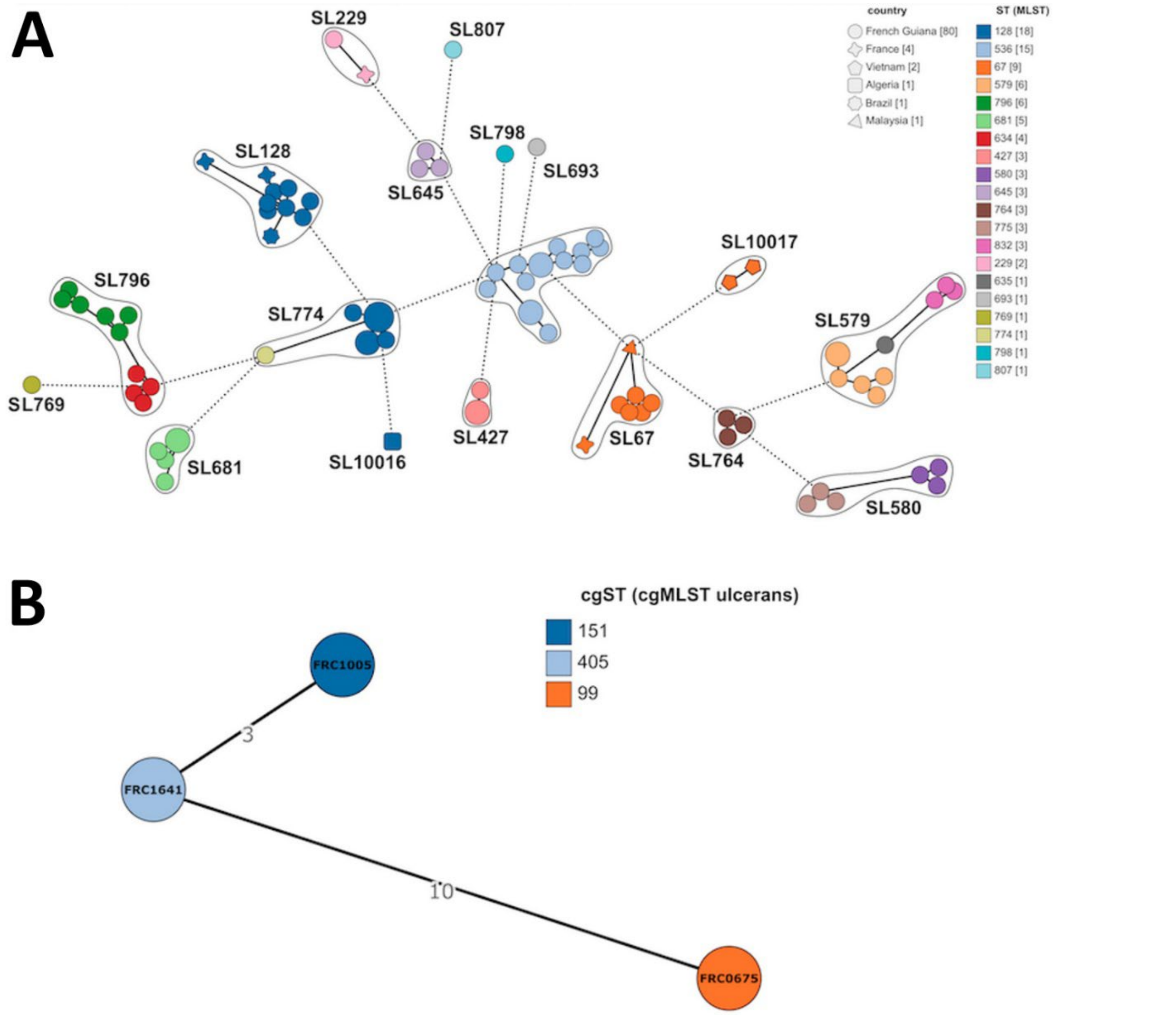

Appendix Figure. Minimum spanning trees of (panel A) *C. diphtheriae* and (panel B) *C. ulcerans* based on cgMLST. *C. diphtheriae* isolates from French Guiana were compared with the most similar isolates from other countries, found in the ENA/GenBank sequence databases, which are represented in different shapes according to country of isolation (see key). cgMLST, core genome multilocus sequence typing; cgST, core genome sequencing. See Institut Pasteur CdSC database (https://bigsdb.pasteur.fr/diphtheria) for futher information.
